# Prevalence and impact of rapid eye movement sleep behavior disorder in multiple system atrophy: a systematic review and meta-analysis

**DOI:** 10.3389/fneur.2024.1453944

**Published:** 2024-10-11

**Authors:** Hui Wang, Ting Zhang, Wenhui Fan

**Affiliations:** Department of Neurology, Sichuan Taikang Hospital, Chengdu, China

**Keywords:** multiple system atrophy, REM sleep behavior disorder, prevalence, motor symptoms, nonmotor symptoms

## Abstract

**Objective:**

Multiple system atrophy (MSA) is commonly associated with rapid eye movement sleep behavior disorder (RBD). Research on the prevalence of RBD in MSA and its effects on MSA patients has yielded inconsistent results. Currently, there is only one meta-analysis discussing the prevalence of RBD in MSA, but no meta-analysis discussing the impact of RBD on MSA.

**Methods:**

A systematic review and meta-analysis was conducted by searching studies related to MSA and RBD in PubMed, Web of Science, Embase, and Cochrane databases. Data were pooled as necessary to calculate prevalence of RBD, odds ratio (OR), weighted mean differences (WMD) with 95% confidence intervals (CI). Heterogeneity was assessed using the *I*^2^ statistic.

**Results:**

The prevalence of polysomnography confirmed-RBD in MSA was 79.9% (95% CI, 68.8–89.3%) in a pooled sample of 598 subjects. Patients with MSA who had RBD were notably younger at examination than those without RBD (WMD −3.26 years, 95% CI −4.99 to −1.53), and the age of disease onset in MSA patients with RBD was significantly lower than in those without RBD (WMD −3.27, 95% CI −5.06 to −1.48). Additionally, RBD was more common among male patients with MSA compared to female patients (OR 2.11, 95% CI 1.31 to 3.39). MSA patients with RBD also exhibited significantly higher Unified Multiple System Atrophy Rating Scale (UMSARS) I and IV scores than those without RBD (WMD 2.99, 95% CI 0.10 to 4.88, and WMD 0.23, 95% CI 0.03 to 0.43).

**Conclusion:**

The prevalence of polysomnography-confirmed RBD in MSA is 79.9%. The prevalence in Asian population was lower than in Europe and America, which might be related to an underestimation in Asian populations. Additionally, patients with MSA and RBD tend to be younger at examination, have an earlier age of onset, and exhibit more severe disease manifestations compared to MSA patients without RBD.

## Introduction

Multiple system atrophy (MSA) is classified as a type of synucleinopathy characterized by symptoms including parkinsonism, autonomic dysfunction, and ataxia (1). A notable non-motor symptom linked to MSA is rapid eye movement behavior disorder (RBD), which involves a lack of muscle atonia and considerable motor activity during rapid eye movement (REM) sleep (2). This condition can result in aggressive and dangerous actions while asleep, along with vivid and intense dreams (2).

Though RBD is relatively uncommon among the general adult populace—affecting 1% but rising to 2–8% in older adults (3)—it seems to occur more frequently in individuals suffering from synucleinopathies such as MSA and Parkinson’s disease (PD) (4). Although there are some research focusing on the occurrence of RBD specifically in MSA patients, numerous studies utilize questionnaires, like the Sleep Behavior Disorder Questionnaire, for diagnosis, which can result in misleading conclusions. The most precise approach for RBD diagnosis involves polysomnography (PSG) (2). A meta-analysis conducted in 2015 indicated an RBD prevalence rate of 88% among MSA patients confirmed via PSG. To delve deeper into this, we performed a meta-analysis of all research published regarding the prevalence of PSG-confirmed RBD in MSA patients, extending up to the year 2024.

Determining the occurrence of RBD among MSA patients and ensuring early detection is vital, as this disorder serves as a potential “red flag” for synucleinopathies (5). Longitudinal studies have indicated that between 17.7 and 65% of those with RBD are likely to develop PD within a timeframe of 5 to 20 years (6, 7). Additionally, individuals diagnosed with both PD and RBD show greater severity in motor disability, cognitive and autonomic dysfunction, extended disease duration, along with a heightened incidence of orthostatic hypotension and visual hallucinations compared to those who do not have RBD (8). Although both PD and MSA are categorized as synucleinopathies, earlier investigations have yet to conclusively show that RBD exacerbates motor symptoms or accelerates disease progression in MSA.

The present study is a comprehensive systematic review and meta-analysis of all published articles on the prevalence of RBD in MSA and its effects on MSA.

## Methods

### Searching strategy

This meta-analysis was conducted following the guidelines of the Preferred Reporting Items for Systematic Reviews and Meta-Analyses (PRISMA) (9). The Cochrane Collaboration definition for systematic review and meta-analysis was strictly followed. Two authors (HW and TZ) independently searched Medline via PubMed, Web of Science, Embase via embase.com, and Cochrane databases for original published studies on the clinical manifestations of MSA patients with or without RBD. Inclusion criteria were studies published in English before March 4, 2024. The search string was as follows: (“Behavior Disorder, REM” OR “Behavior Disorders, REM” OR “REM Behavior Disorders” OR “REM Behavior Disorder” OR “Behavior Disorder, Rapid Eye Movement Sleep” OR “Rapid Eye Movement Sleep Behavior Disorder”) AND (“Atrophy, Multiple System” OR “Multiple System Atrophies” OR “Multisystemic Atrophy” OR “Atrophies, Multisystemic” OR “Atrophy, Multisystemic” OR “Multisystemic Atrophies” OR “Multiple System Atrophy Syndrome” OR “Multisystem Atrophy” OR “Atrophies, Multisystem” OR “Atrophy, Multisystem” OR “Multisystem Atrophies”).

### Study selection criteria

Articles were initially screened based on their titles and abstracts, with full text consulted when necessary. Patients were diagnosed with PSG-confirmed RBD according to the International Classification of Sleep Disorders, 3rd Edition (ICSD-3), which requires detection of REM sleep without atonia and episodes of vocalization and/or motor behavior during REM sleep during polysomnography (2). The episodes of vocalization or motor behaviors can also be based on clinical history.

We recorded demographic characteristics for all selected studies. “‘Disease onset’ was defined as the first appearance of any motor symptom (e.g., parkinsonism symptoms or cerebellar ataxia) or selected autonomic features (orthostatic hypotension or neurogenic bladder dysfunction).”

Inclusion criteria were: (1) original data on RBD and clinical symptoms of MSA, (2) patients diagnosed with probable MSA, classification into those with or without RBD, (3) and sufficient data to calculate differences in the incidence or severity of motor symptoms of MSA.

Exclusion criteria were: (1) reviews, editorials, conference abstracts, or case reports; (2) focus solely on RBD characteristics, pathogenic mechanisms, or MSA management with RBD; (3) comparisons between MSA and other synucleinopathies; (4) insufficient data for meta-analysis; (5) non-English articles; (6) or studies not involving human subjects. Discrepancies in article inclusion were resolved by a third author (WF).

### Data extraction and study quality assessment

The data extracted from the original articles included the surname of the first author, publication year, country, sample size, method of RBD assessment, prevalence of RBD, mean age of patients, sex, disease onset, and score on the Unified Multiple System Atrophy Rating Scale (UMSARS). For longitudinal studies, only baseline data were extracted.

The quality of the included studies was assessed using the guidelines from the Newcastle–Ottawa Scale (10). Any discrepancies were resolved through consensus among all authors.

### Statistical analysis

The STATA software version 16.0 was used for statistical analysis. Odds ratio (OR), weighted mean difference (WMD), or standardized mean difference (SMD) with 95% confidence intervals (95% CI) were used to report pooled results on dichotomous and continuous variables. A *p*-value equal to or less than 0.05 was considered statistically significant. The primary outcome measure was frequency of RBD in MSA as reported in prevalence (%). The pooled prevalence of RBD and 95% confidence intervals were obtained by using a DerSimonian–Laird random-effects model with double arcsine transformation (11). Meta-regression analyses were used to assess the statistical results of whether disease duration affects the statistical results of the incidence of RBD in MSA patients. The heterogeneity across studies was evaluated using Cochrane’s *I*^2^ values. *I*^2^ > 75% was defined as high heterogeneity, 50% < *I*^2^ < 75% as moderate heterogeneity, 25% < *I*^2^ < 50% as low heterogeneity, and *I*^2^ < 25% as homogeneity. We used a fixed-effects model to meta-analyze data showing homogeneity and low heterogeneity, and a random-effects model to analyze data classified as moderate or high heterogeneity. We used the one by one elimination method on STATA to perform sensitivity analyses to detect potential sources of heterogeneity, and a funnel plot and Egger and Begg’s test was created to detect publication biases.

## Results

**Figure 1:**
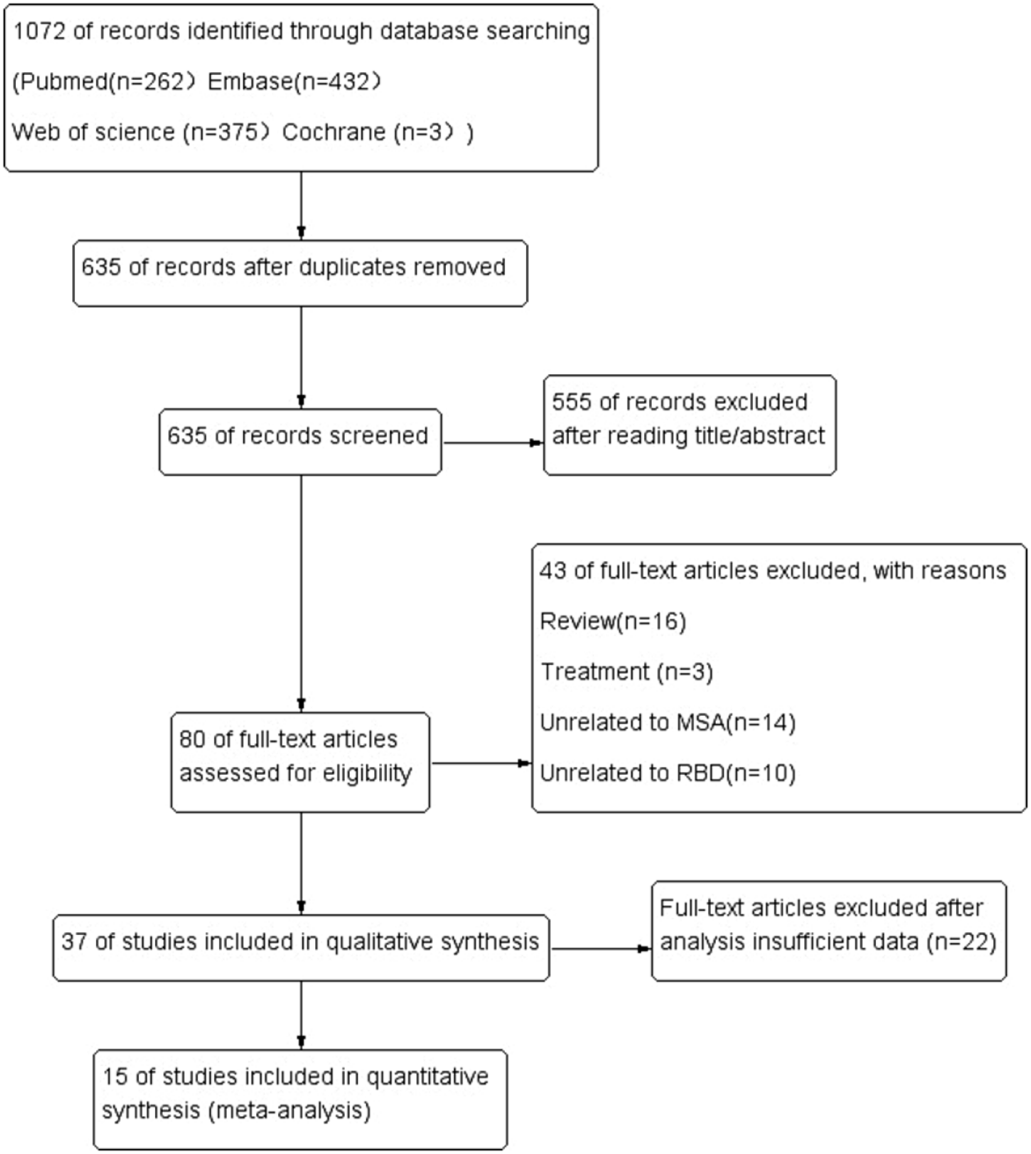
Flow diagram of systematic literature searching.

The literature search yielded 1,072 potentially relevant articles (Figure 1). After eliminating duplicates, 635 records were reviewed and 555 were excluded during the title and abstract screening phase. The remaining 80 full-text articles were assessed for eligibility, and 54 were excluded because they were reviews (*n* = 16), studies about treatment (*n* = 3), studies unrelated MSA (*n* = 14) or RBD (*n* = 10), and insufficient date (*n* = 22).

In the end, 15 articles were ultimately included in our review, involving a total of 817 MSA patients. Six original researches were performed in Asia, 4 in North America, and 5 in Europe. The meta-analysis was conducted for all MSA patients defined by prevalence of RBD, age, disease onset, and score on the UMSARS.

### Prevalence of PSG-confirmed RBD

**Figure 2:**
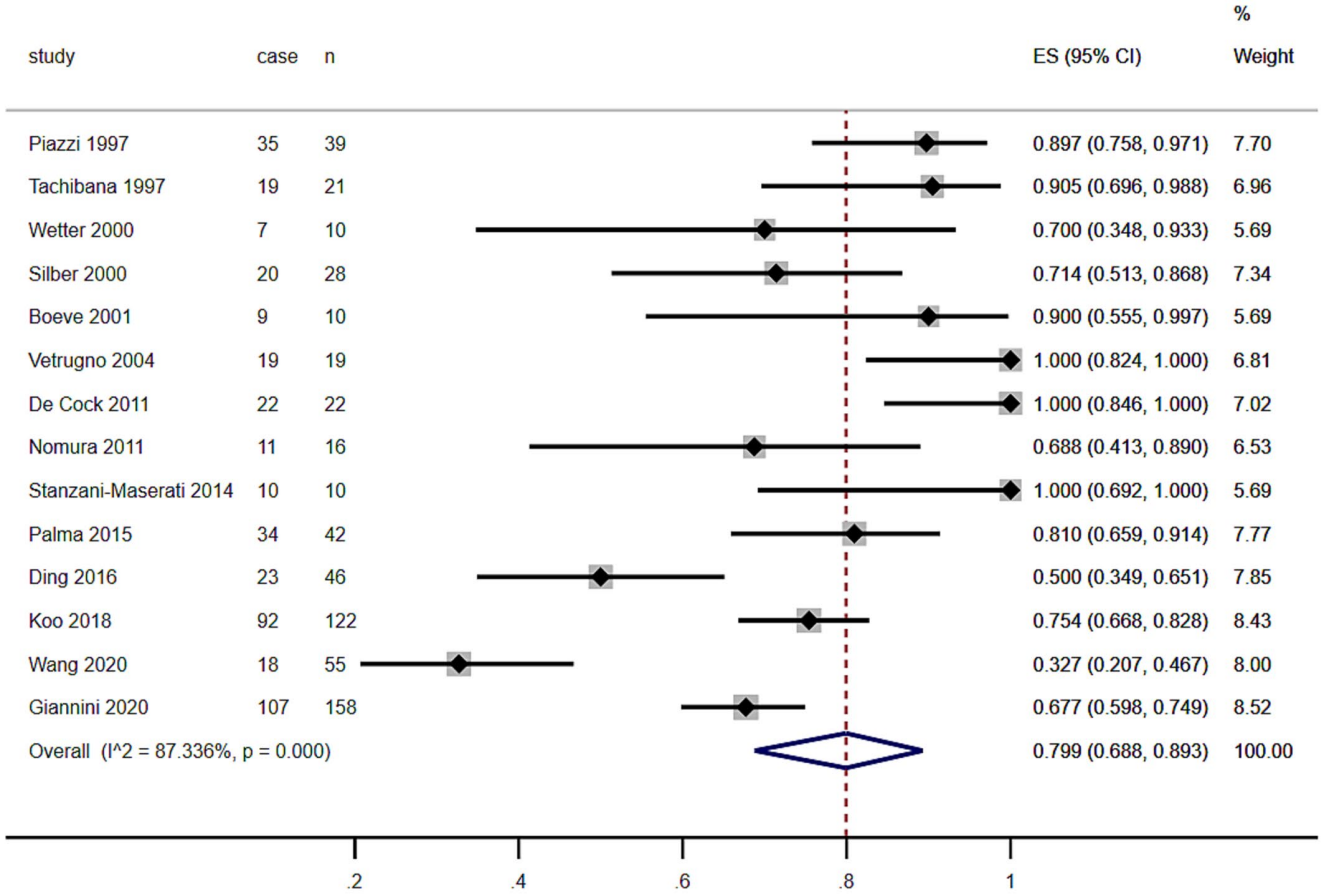
Forest plot on the pooled prevalence of rapid eye movement sleep behavior disorder in multiple system atrophy.

As shown in the Table 1 and Figure 2, including the results of the present study, the summary prevalence of PSG-confirmed RBD in MSA was 79.9% (95% CI, 68.8–89.3%) in a pooled sample of 598 subjects. There was no evidence of publication bias as Egger’s test was not significant (Supplementary Figure S1.1).

**Table 1:** Characteristics of studies included in the meta-analyses.

Reference	First author	Year	Country	Sample size	Diagnostic RBD method	Proportion of RBD (%)	Disease duration (year)	NOS score
(22)	Plazzi	1997	Italy	39	PSG	90	N	5
(23)	Tachibana	1997	Japan	21	PSG	90.5	2.5	5
(24)	Wetter	2000	Germany	10	PSG	70	4	6
(25)	Silber	2000	USA	28	PSG	71	N	7
(14)	Boeve	2001	USA	10	PSG	90	N	6
(15)	Vetrugno	2004	Italy	19	PSG	100	N	7
(26)	De Cock	2011	USA	22	PSG	100	5.5	6
(27)	Nomura	2011	Japan	16	PSG	68.8	4.7	7
(28)	Stanzani-Maserati	2014	Italy	10	PSG	100	5	6
(12)	Palma	2015	USA	42	PSG	80.9	3.2	7
(18)	Ding	2016	China	46	PSG	50	1.5	6
(29)	Koo	2018	South Korea	122	PSG	75.4	N	7
(13)	Wang	2020	China	55	PSG	32.7	7	7
(30)	Giannini	2020	Italy	158	PSG	67.7	2.66	7
Total				598	PSG	79.9		

PSG, polysomnography; RBD, REM sleep behavior disorder; MSA, multiple system atrophy; NOS, Newcastle–Ottawa Scale. N indicates that this study has no relevant data.

**Figure 3:**
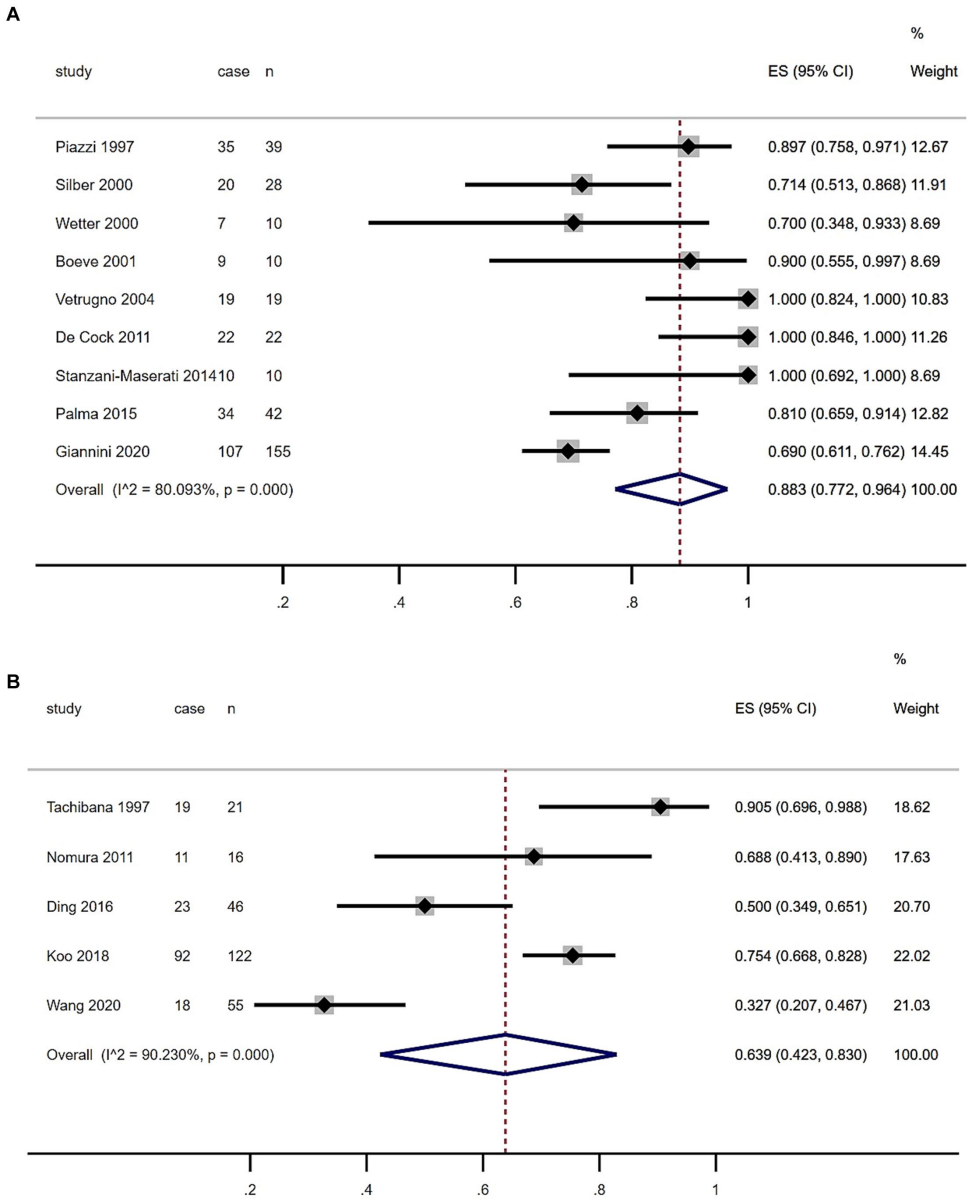
Forest plot of rapid eye movement sleep behavior disorder prevalence among multiple system atrophy patients in European, US **(A)** and Asian **(B)** populations.

As shown in the Table 1 and Figure 3, the summary prevalence of PSG-confirmed RBD of MSA in Europe and USA was 88.3% (95% CI, 77.2–96.4%), which was higher than that in Asia (63.9, 95% CI, 42.3.7–83.0%). There was no evidence of publication bias as Egger’s test was not significant (Supplementary Figures S1.2, 1.3).

**Figure 4:**
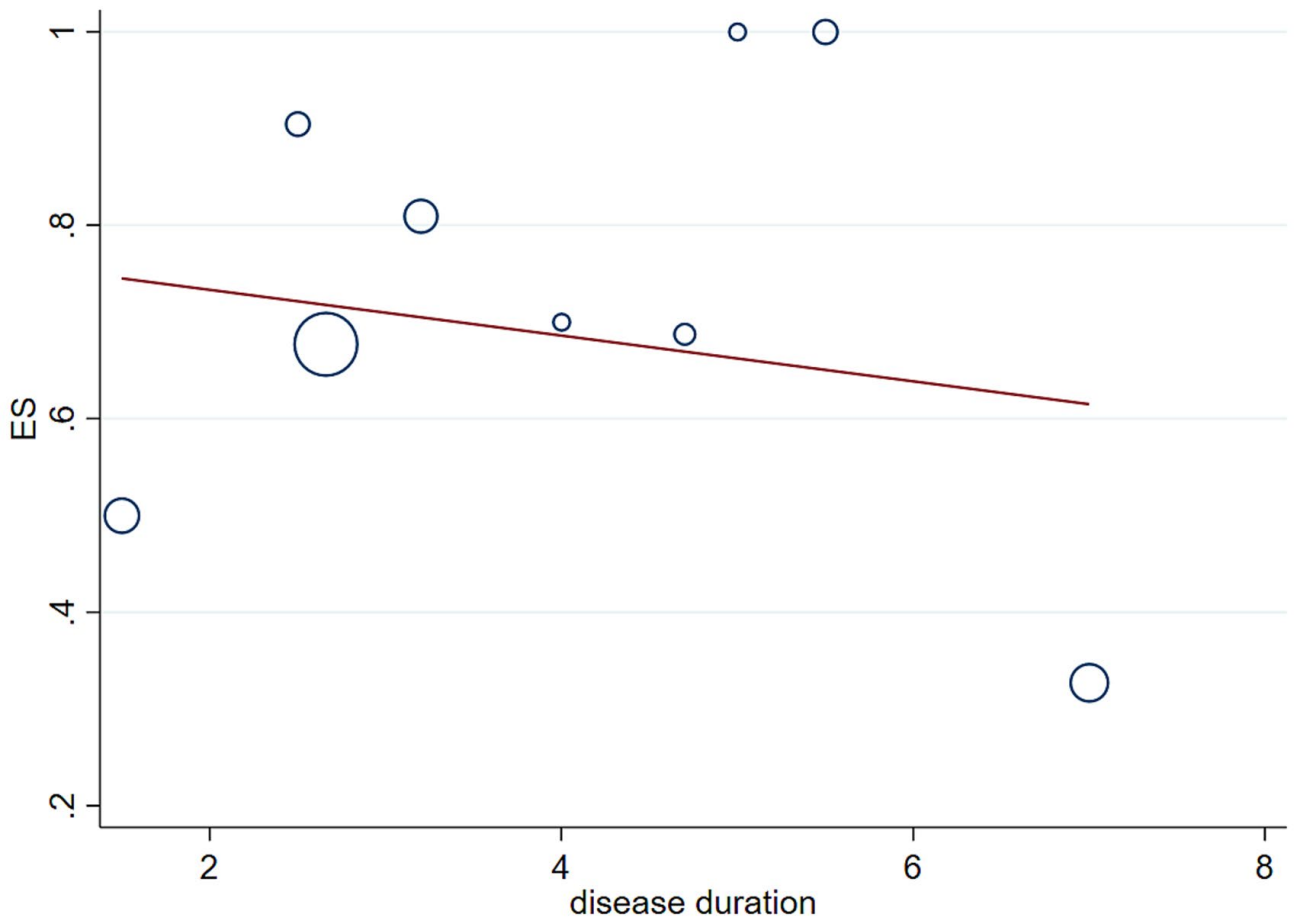
Meta-regression modeling of RBD prevalence in MSA patients adjusted for disease duration.

We performed meta-regression analyses to assess whether disease duration affects the statistical results of the incidence of RBD in patients with MSA, and the results showed that disease duration in patients with MSA does not affect the statistical results of the incidence of RBD in MSA patients (*p* = 0.628, Figure 4).

### Differences in age, gender, and disease onset between MSA patients with or without RBD

The rarity of MSA has resulted in a limited number of studies investigating the effects of RBD on both motor and non-motor symptoms associated with MSA. So far, only 2 relevant studies were identified, which employed by PSG for the diagnosis of RBD.

**Figure 5:**
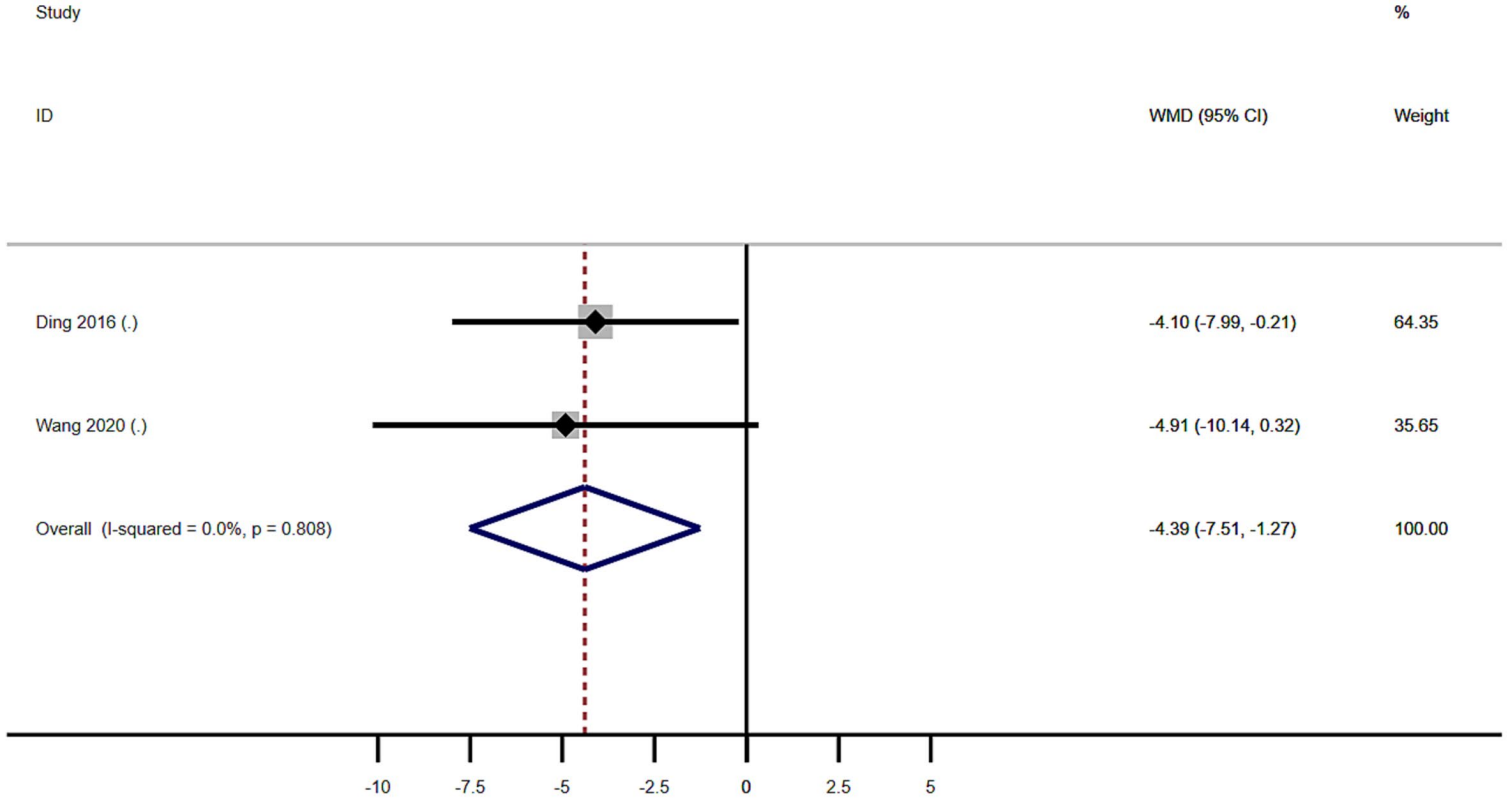
Forest plot showing weighted mean difference (WMD) in age between multiple system atrophy patients with or without rapid eye movement sleep behavior disorder.

Subgroup analysis based on age included 101 patients (Table 2). MSA patients with RBD were significantly younger at examination than those without RBD (WMD −4.39 years, 95% CI −7.51 to −1.27; *I*^2^ = 0%; Table 2 and Figure 5). Begg’s test did not show significant publication bias (Supplementary Figure S2).

**Table 2:** Characteristics of studies included in the meta-analyses.

Reference	First author	Year	Country	Sample size	Diagnostic MSA method	Mean age of patients with RBD/without RBD	Proportion of male patients with/without RBD (%)	NOS score
(18)	Ding	2016	China	46	SCS (2008)	52.6 ± 7.4/56.7 ± 6	78.3/65.2	6
(13)	Wang	2020	China	55	SCS (2008)	59.5 ± 8.1/64.4 ± 11.3	72.2/59.5	7

MSA, multiple system atrophy; NOS, Newcastle–Ottawa Scale (10); PSG, polysomnography; RBD, REM sleep behavior disorder; SCS, second consensus statement on the diagnosis of multiple system atrophy in 2008 (1).

**Figure 6:**
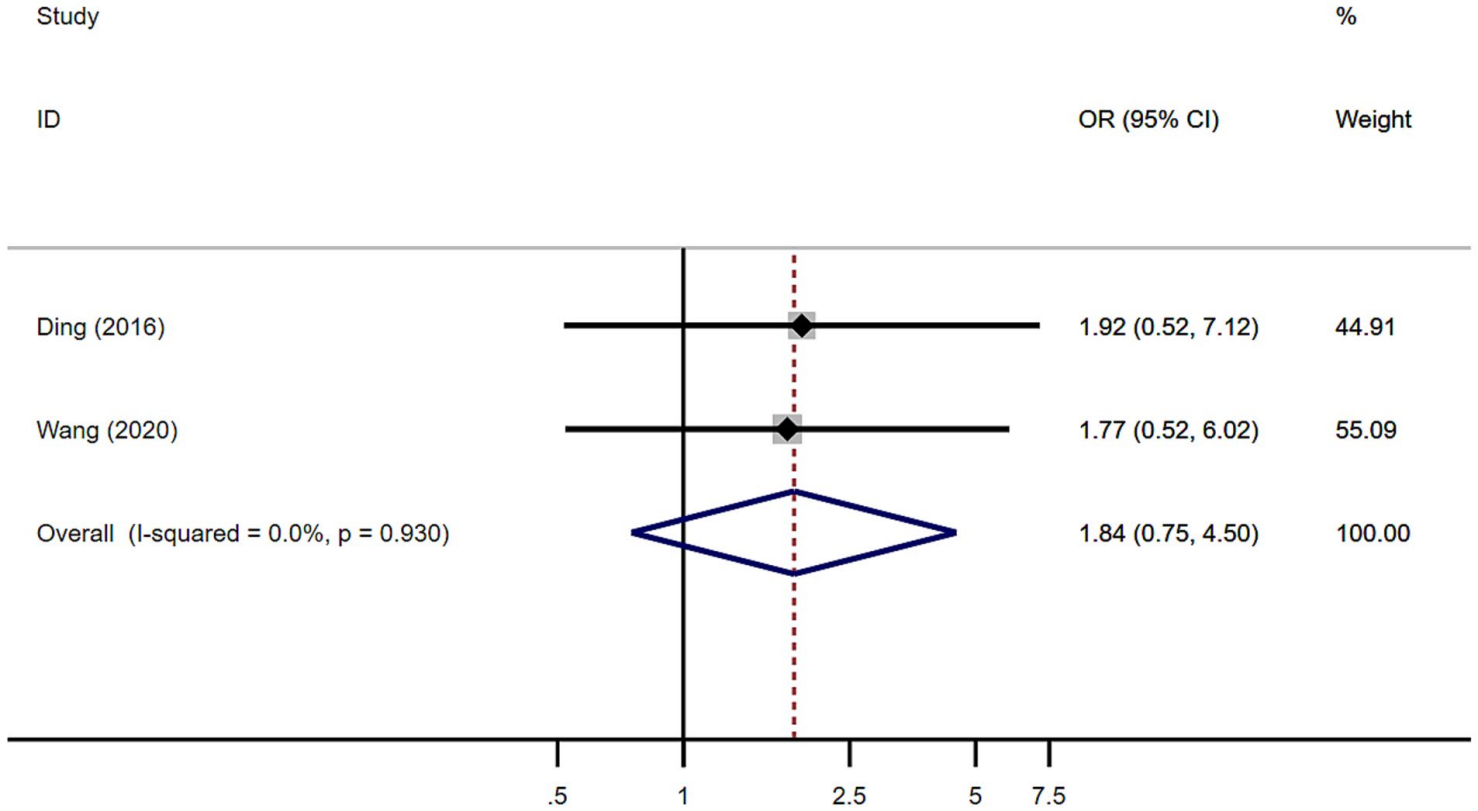
Forest plot showing odds ratio (OR) in sex between multiple system atrophy patients with or without rapid eye movement sleep behavior disorder.

A total of 101 patients with MSA from 2 studies were included in analysis by sex. There was no significant difference in sex between MSA patients with or without RBD (Figure 6). Our study showed homogeneity (*I*^2^ = 0%). Begg’s test did not show significant publication bias (Supplementary Figure S3).

**Figure 7:**
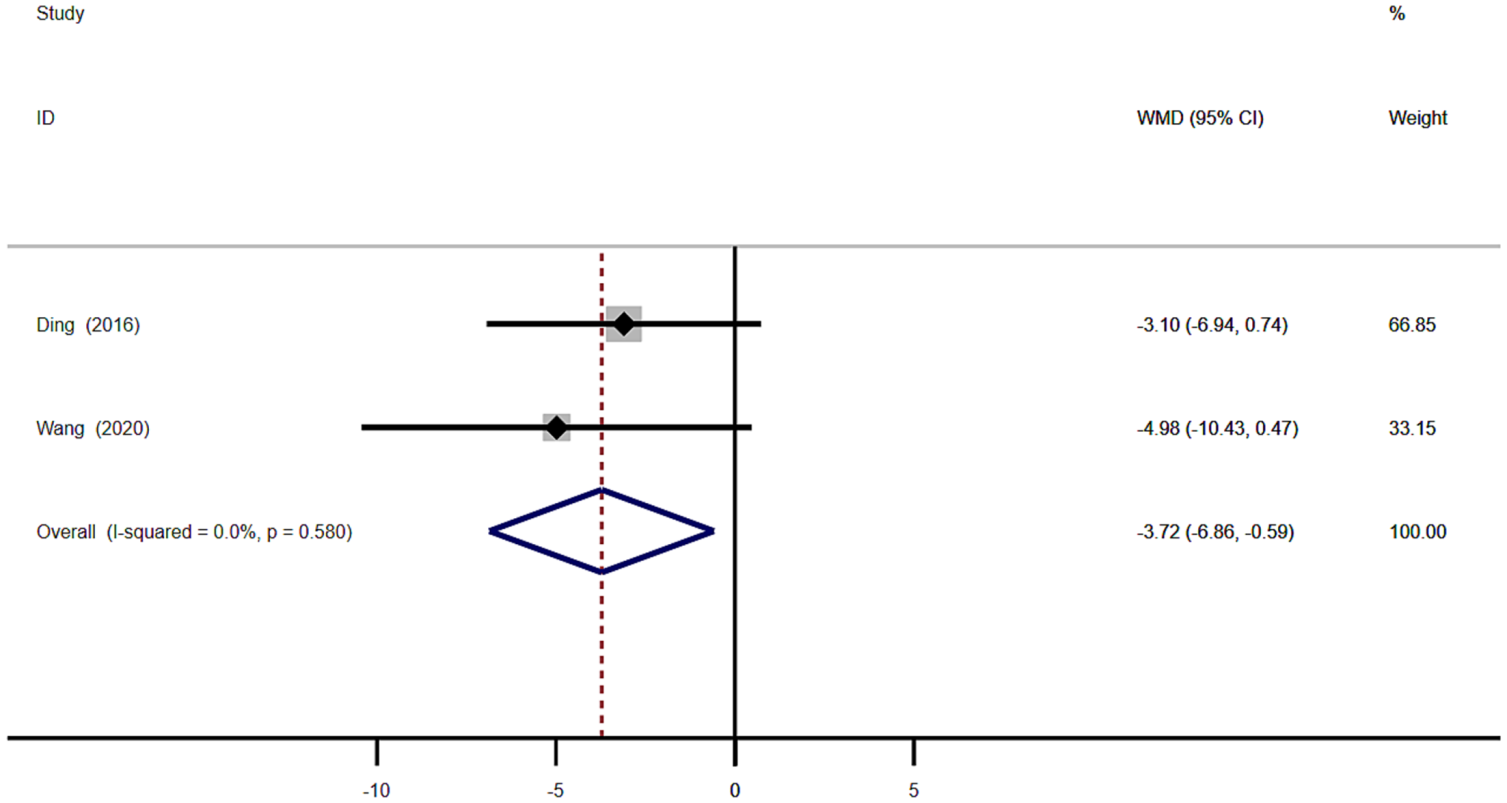
Forest plot showing weighted mean difference (WMD) in disease onset age between multiple system atrophy patients with or without rapid eye movement sleep behavior disorder.

Meta-analysis of the age of onset in 101 patients with MSA from 2 studies showed that the age of disease onset in MSA patients with RBD was significantly lower than in MSA patients without RBD (WMD −3.72, 95% CI −6.86 to −0.59; Figure 7). This study showed homogeneity (*I*^2^ = 0%). Begg’s test did not show significant publication bias (Supplementary Figure S4).

### Differences in UMSARS scores between MSA patients with or without RBD

A total of 101 patients with MSA from 2 studies were included in analysis by UMSARS part I, II, and IV sections scores.

**Figure 8:**
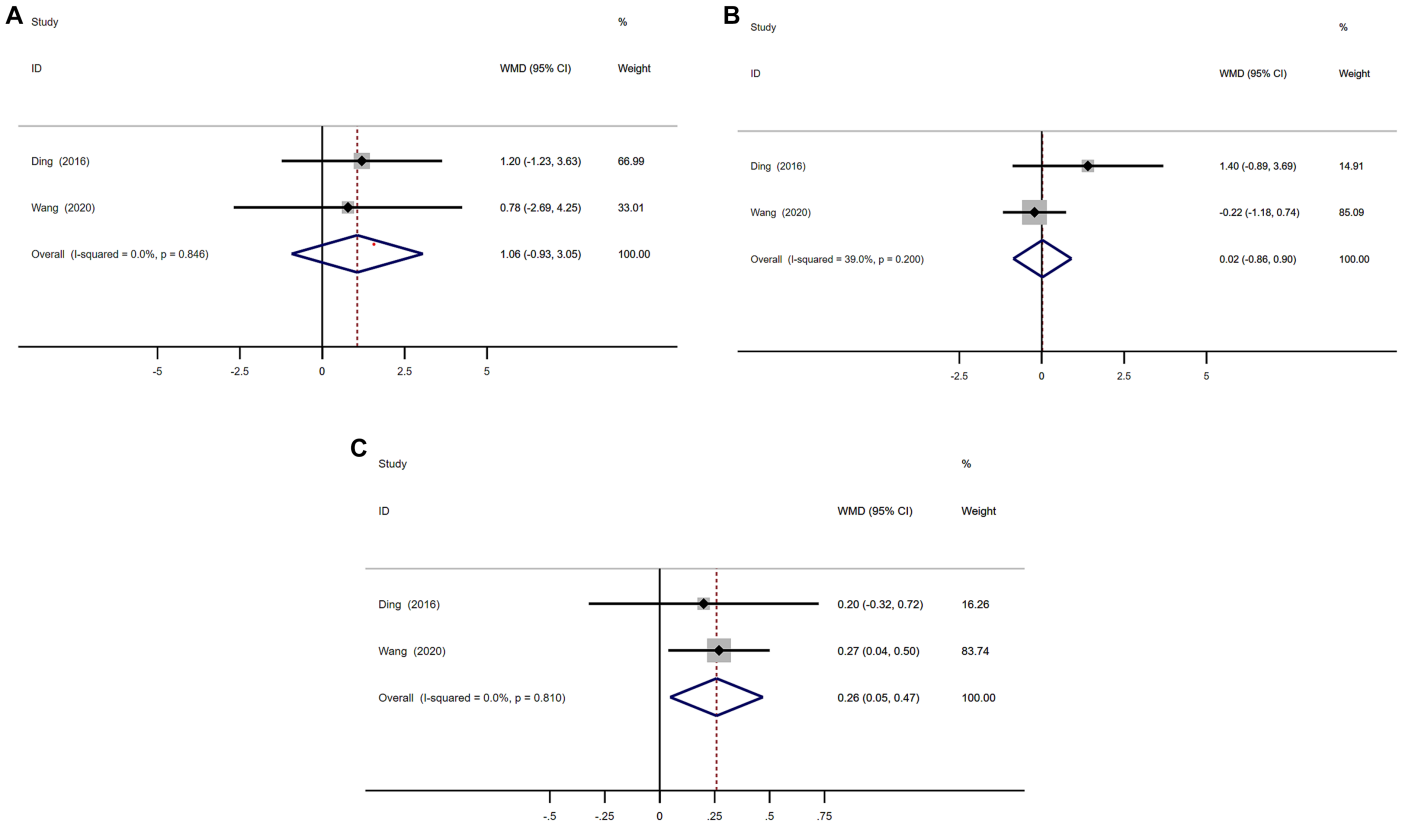
Forest plot showing weighted mean difference (WMD) in UMSARS I, II, and IV sections scores **(A–C)** between multiple system atrophy patients with or without rapid eye movement sleep behavior disorder.

There were no significant difference in UMSARS I and II scores between MSA patients with or without RBD (Figures 8A,B). The heterogeneity of this study was low (*I*^2^ = 0 and 39.0%). There was no evidence of publication bias as Begg’s test was not significant (Supplementary Figure S5; Figure 7).

MSA with RBD group had significant higher scores on UMSARS Part IV than MSA without RBD group (WMD 0.26, 95% CI 0.05 to 0.47; *I*^2^ = 0%; Figure 8C). There was no evidence of publication bias as Begg’s test was not significant (Supplementary Figure S7).

## Discussion

The meta-analysis conducted in this study revealed a striking prevalence rate of PSG-confirmed RBD in patients with MSA, recorded at 79.9% with a 95% confidence interval ranging from 68.8 to 89.3% (Figure 2). Notably, this figure contrasts with findings from a previous study published in 2015, which reported a prevalence rate of 88% (12). This difference in prevalence rates may be significantly influenced by the demographic composition of the patient populations studied. The 2015 research primarily focused on individuals from European and North American backgrounds, while the current meta-analysis, covering the years 2015 to 2024, included a diverse group of participants predominantly from various Asian regions (Table 1). The geographical and cultural factors associated with these populations could therefore play a critical role in shaping the overall prevalence of RBD observed in this analysis. Earlier studies have suggested that the prevalence of RBD among MSA patients tends to be lower within Asian populations in comparison to their European counterparts (13). The contributing factors to this disparity may extend beyond genetic differences; inconsistencies in diagnostic criteria across regions could also impact reported prevalence rates. For example, some regions only considered REM sleep without atonia (RSWA) or dream interpretation for RBD diagnosis, leading to potentially inflated prevalence estimates compared to regions that considered both criteria (14, 15). Additionally, findings from recent research indicated that Chinese patients demonstrating RBD characteristics exhibited lower electromyographic activity relative to patients of other ethnicities (16), which could hinder their ability to meet the diagnostic benchmarks for RBD even when they clearly exhibit specific behaviors during REM sleep. A previous study highlighted the dilemma faced by 22 patients with MSA who presented with significant behavioral disturbances during REM sleep, as indicated by their scores of ≥5 on The Sleep Behavior Disorder Questionnaire (RBDSQ) (13, 17). However, despite these scores, only 10 of those individuals fulfilled the diagnostic requirements for PSG-confirmed RBD. Our meta-analysis indicated that the prevalence rates of RBD in MSA patients were notably higher in Europe and North America (88.3%) compared to those in Asia (63.9%) (Figure 3). Such findings suggest that there may be an underestimation of RBD prevalence among Asian patients with MSA, which could ultimately result in underdiagnosis. This underdiagnosis may stem from inconclusive electromyographic findings on PSG, despite the presence of prominent clinical symptoms. To address these discrepancies, we advocate for the development of new diagnostic criteria for RBD specifically tailored to the unique characteristics of Asian populations.

We performed a meta-analysis of age, sex, age of onset, and the severity of motor symptoms in MSA patients with or without RBD. The result showed MSA patients with RBD were significantly younger than those without RBD (WMD −4.39 years, 95% CI −7.51 to −1.27; Figure 5) and the age of disease onset in MSA patients with RBD was significantly lower than in MSA patients without RBD (WMD −3.72, 95% CI −6.86 to −0.59; Figure 7), and MSA patients with RBD scored higher on the UMSARS score, especially in parts IV (WMD 0.26, 95% CI 0.05 to 0.47; *I*^2^ = 0%; Figure 8C). It is worth noting that the studies we have reviewed are all from Asia, with no published studies from Europe and the United States on the effects of RBD on MSA. Given potential ethnic differences, results from Europe and the United States may vary from those in Asia. Further studies from Europe and the United States are necessary to validate our findings.

Previous studies have indicated that individuals with MSA and RBD tend to be younger and experience an earlier onset of the disease compared to those without RBD. While previous studies did not find this difference to be statistically significant (13, 18), this meta-analysis combines data from various studies and demonstrates a statistically significant variance. This implies that individuals with MSA and RBD may indeed have an earlier onset of the disease compared to those without RBD. Although both MSA and PD are synucleinopathies, they also have many differences, and previous studies have shown that PD patients with RBD are older than those without RBD (8, 19).

Previous studies have indicated a higher prevalence of male patients with MSA with RBD, although the difference was not deemed statistically significant (13, 18–20). Through a meta-analysis of various relevant studies, it was determined that RBD was more common among male MSA patients compared to female patients, the difference was not statistically significant as well (Figure 6). Previous study showed that males in the general population showed a trend for a higher risk of probable/possible RBD, especially among the male older adults (aged ≥60) (21).

Our meta-analysis revealed that MSA patients with RBD had higher UMSARS scores compared to those without RBD, with statistically significant differences observed in parts IV of the UMSARS scale (Figure 8C). Previous studies have also reported higher UMSARS scores in MSA patients with RBD, although the differences were not statistically significant, likely due to the rarity of MSA and small sample sizes (13, 18–20). Furthermore, our findings suggest that MSA patients with RBD were younger, had earlier disease onset, and exhibited more severe symptoms, indicating a potential acceleration of disease progression and symptom exacerbation. Previous research has identified RBD as a ‘red flag’ for faster disease progression and increased severity in synucleinopathies, including PD and MSA (5). PD patients with RBD demonstrate more severe motor disability, cognitive and autonomic impairment, higher prevalence of orthostatic hypotension, and visual hallucinations compared to those without RBD (8, 19).

Several limitations should be acknowledged in this study. First, MSA is a rare disease, leading to a scarcity of research on the correlation between RBD and MSA with limited sample sizes. Further studies with larger sample sizes are necessary to confirm our findings. Second, considering the present study discussion, it is possible that the prevalence of RBD in Asian MSA patients is underestimated, potentially affecting the accuracy of our statistics on RBD prevalence in MSA. Third, all of the studies we have reviewed on the effects of RBD on MSA have originated from Asia, with no published studies on the effects of RBD on MSA from Europe and the United States. This lack of diversity in geographical representation raises questions about potential ethnic differences in the effects of RBD on MSA. Fourth, due to the limited number of published studies examining the effects of RBD on both motor and non-motor activities in MSA, we expanded our sample size by incorporating two studies that utilized questionnaires for the diagnosis of RBD. This inclusion may have influenced the outcomes of the meta-analysis. Fifth, this study did not register systematic review protocol, which may lead to reporting bias. Sixth, our meta-analysis exclusively included published articles in English. As a result, many non-English articles were omitted from the statistical analysis. Additionally, articles reporting a low percentage of RBD in MSA patients may face challenges in publication, often being limited to abstracts, which we also did not include. This exclusion may impact the our statistical results. Finally, the diagnostic criteria for MSA in the literature included were based on the most recent criteria available at the time of publication. As diagnostic criteria for MSA are continuously evolving, this may result in inaccuracies when diagnosing patients with MSA in older literature compared to more recent publications.

## Conclusion

The prevalence of polysomnography-confirmed RBD in MSA is 79.9%. The prevalence in Asian population was lower than in Europe and America, which might be related to an underestimation in Asian populations. Additionally, patients with MSA and RBD tend to be younger at examination, have an earlier age of onset, and exhibit more severe disease manifestations compared to MSA patients without RBD.

## Data Availability

The original contributions presented in the study are included in the article/Supplementary material, further inquiries can be directed to the corresponding authors.
