# Supplementary material for: Prevalence and impact of rapid eye movement sleep behavior disorder in multiple system atrophy: a systematic review and meta-analysis
Source: Front Neurol. 2024 Oct 11;15:1453944. doi: 10.3389/fneur.2024.1453944 (PMC11502359; doi:10.3389/fneur.2024.1453944)
Supplement: Supplementary file 1 [file Data_Sheet_1.DOCX]

**Cochrane:**

Search Name:

Date Run: 06/03/2024 17:45:26

Comment:

ID Search Hits

#1 MeSH descriptor: [REM Sleep Behavior Disorder] explode all trees 48

#2 (Rem sleep behavior disorder):ti,ab,kw OR (Behavior Disorder, REM):ti,ab,kw OR (Behavior Disorders, REM):ti,ab,kw OR (REM Behavior Disorders):ti,ab,kw OR (REM Behavior Disorder):ti,ab,kw 226

#3 (Behavior Disorder, Rapid Eye Movement Sleep):ti,ab,kw OR (Rapid Eye Movement Sleep Behavior Disorder):ti,ab,kw 76

#4 #1 OR #2 OR #3 244

#5 MeSH descriptor: [Multiple System Atrophy] explode all trees 171

#6 (Multiple System Atrophy):ti,ab,kw OR (Atrophy, Multiple System):ti,ab,kw OR (Multiple System Atrophies):ti,ab,kw OR (Multisystemic Atrophy):ti,ab,kw OR (Atrophies, Multisystemic):ti,ab,kw 492

#7 (Atrophy, Multisystemic):ti,ab,kw OR (Multisystemic Atrophies):ti,ab,kw OR (Multiple System Atrophy Syndrome):ti,ab,kw OR (Multisystem Atrophy):ti,ab,kw OR (Atrophies, Multisystem):ti,ab,kw 240

#8 (Atrophy, Multisystem):ti,ab,kw OR (Multisystem Atrophies):ti,ab,kw 15

#9 #5 OR #6 OR #7 OR #8 523

#10 #4 AND #9 3

**Embase**

('rem sleep behavior disorder'/exp OR 'rem sleep behavior disorder' OR 'behavior disorder, rem' OR 'behavior disorders, rem' OR 'rem behavior disorders'/exp OR 'rem behavior disorders' OR 'rem behavior disorder'/exp OR 'rem behavior disorder' OR 'behavior disorder, rapid eye movement sleep' OR 'rapid eye movement sleep behavior disorder'/exp OR 'rapid eye movement sleep behavior disorder') AND ('multiple system atrophy':ab,ti OR 'atrophy, multiple system':ab,ti OR 'multiple system atrophies':ab,ti OR 'multisystemic atrophy':ab,ti OR 'atrophies, multisystemic':ab,ti OR 'atrophy, multisystemic':ab,ti OR 'multisystemic atrophies':ab,ti OR 'multiple system atrophy syndrome':ab,ti OR 'multisystem atrophy':ab,ti OR 'atrophies, multisystem':ab,ti OR 'atrophy, multisystem':ab,ti OR 'multisystem atrophies':ab,ti)-----432

**Pubmed:**

((((((((Behavior Disorder, REM[Title/Abstract]) ) OR (Behavior Disorders, REM[Title/Abstract])) OR (REM Behavior Disorders[Title/Abstract])) OR (REM Behavior Disorder[Title/Abstract])) OR (Behavior Disorder, Rapid Eye Movement Sleep[Title/Abstract])) OR (Rapid Eye Movement Sleep Behavior Disorder[Title/Abstract])) OR ("REM Sleep Behavior Disorder"[Mesh])) AND (("Multiple System Atrophy"[Mesh]) OR ((((((((((((multiple system atrophy[Title/Abstract]) OR (Atrophy, Multiple System[Title/Abstract])) OR (Multiple System Atrophies[Title/Abstract])) OR (Multisystemic Atrophy[Title/Abstract])) OR (Atrophies, Multisystemic[Title/Abstract])) OR (Atrophy, Multisystemic[Title/Abstract])) OR (Multisystemic Atrophies[Title/Abstract])) OR (Multiple System Atrophy Syndrome[Title/Abstract])) OR (Multisystem Atrophy[Title/Abstract])) OR (Atrophies, Multisystem[Title/Abstract])) OR (Atrophy, Multisystem[Title/Abstract])) OR (Multisystem Atrophies[Title/Abstract])))----261

**Web of Science:**

#1=((((((TS=(Rem sleep behavior disorder)) OR TS=(Behavior Disorder, REM)) OR TS=(Behavior Disorders, REM)) OR TS=(REM Behavior Disorders)) OR TS=(REM Behavior Disorder)) OR TS=(Behavior Disorder, Rapid Eye Movement Sleep)) OR TS=(Rapid Eye Movement Sleep Behavior Disorder)

#2=(((((((((((TS=(Atrophy, Multiple System)) OR TS=(Atrophy, Multiple System)) OR TS=(Multiple System Atrophies)) OR TS=(Multisystemic Atrophy)) OR TS=(Atrophies, Multisystemic)) OR TS=(Atrophy, Multisystemic)) OR TS=(Multisystemic Atrophies)) OR TS=(Multiple System Atrophy Syndrome)) OR TS=(Multisystem Atrophy)) OR TS=(Atrophies, Multisystem)) OR TS=(Atrophy, Multisystem)) OR TS=(Multisystem Atrophies)

#3=#1 AND #2-----------375
