# Supplementary material for: Prevalence and impact of rapid eye movement sleep behavior disorder in multiple system atrophy: a systematic review and meta-analysis
Source: Front Neurol. 2024 Oct 11;15:1453944. doi: 10.3389/fneur.2024.1453944 (PMC11502359; doi:10.3389/fneur.2024.1453944)
Supplement: Supplementary file 2 [file Data_Sheet_2.PDF]

Figure 1.1

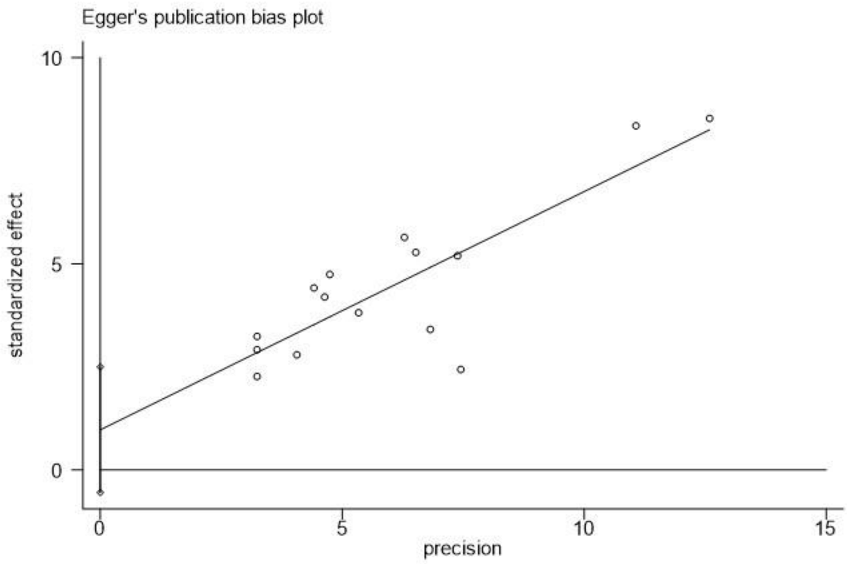

Egger's test

Std_Eff	Coef.	Std. Err.	t	P>|t|	[95% Conf.	Interval]
slope	.5778137	.1062204	5.44	0.000	.3483385	.807289
bias	.9746117	.7043195	1.38	0.190	-.5469782	2.496202

Figure 1.2

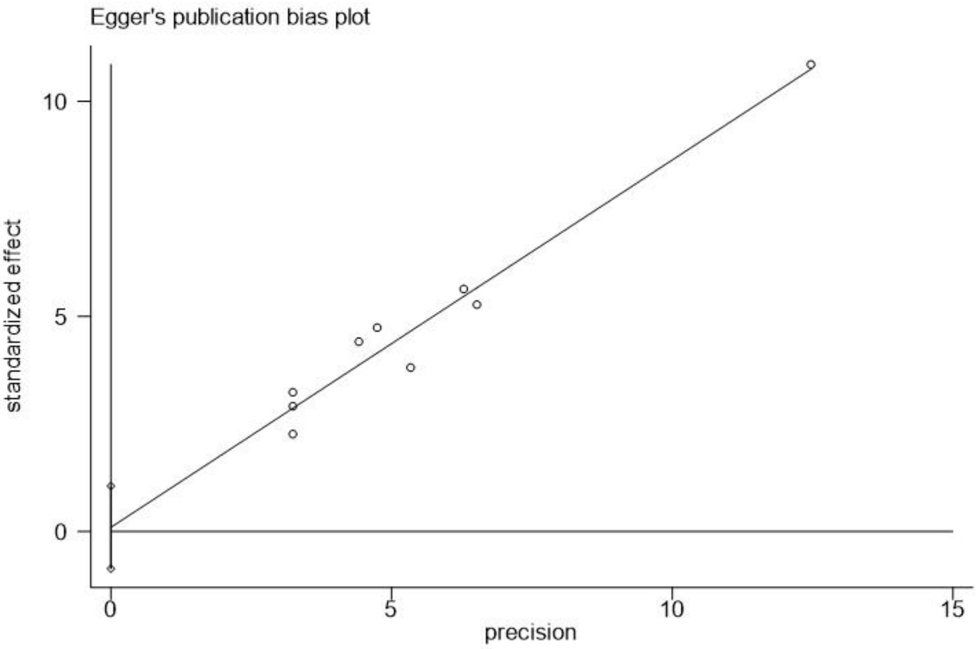

Egger's test

Std_Eff	Coef.	Std. Err.	t	P>|t|	[95% Conf.	Interval]
slope	.8542004	.0660842	12.93	0.000	.6979361	1.010465
bias	.0999147	.4058786	0.25	0.813	-.8598357	1.059665

Figure 1.3

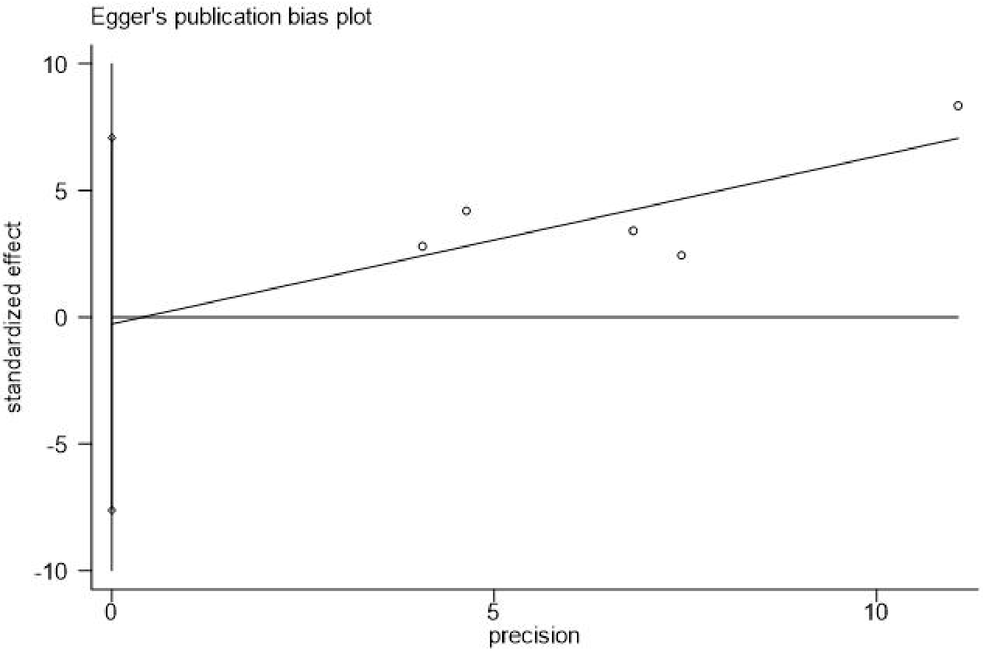

Egger's test

Std_Eff	Coef.	Std. Err.	t	P>|t|	[95% Conf.	Interval]
slope	.6620012	.318732	2.08	0.129	-.3523464	1.676349
bias	-.2699661	2.309433	-0.12	0.914	-7.619612	7.07968

Figure 2

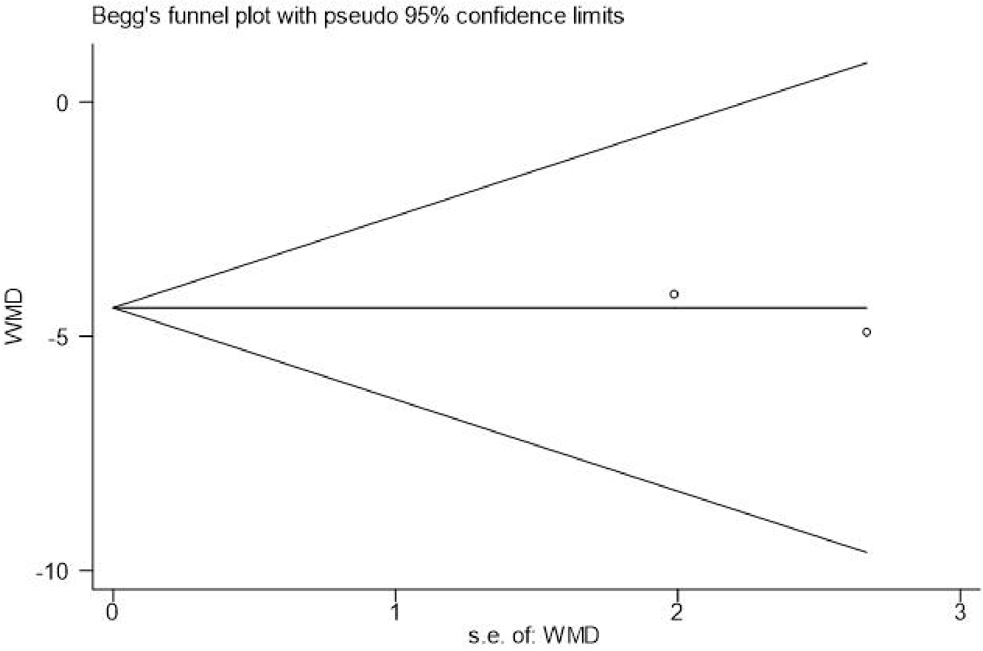


```
Begg's Test

  adj. Kendall's Score (P-Q) =       -1
          Std. Dev. of Score =     1.00
           Number of Studies =        2
                          z  =    -1.00
                    Pr > |z| =    0.317
                          z  =     0.00 (continuity corrected)
                    Pr > |z| =    1.000 (continuity corrected)
```

Figure 3

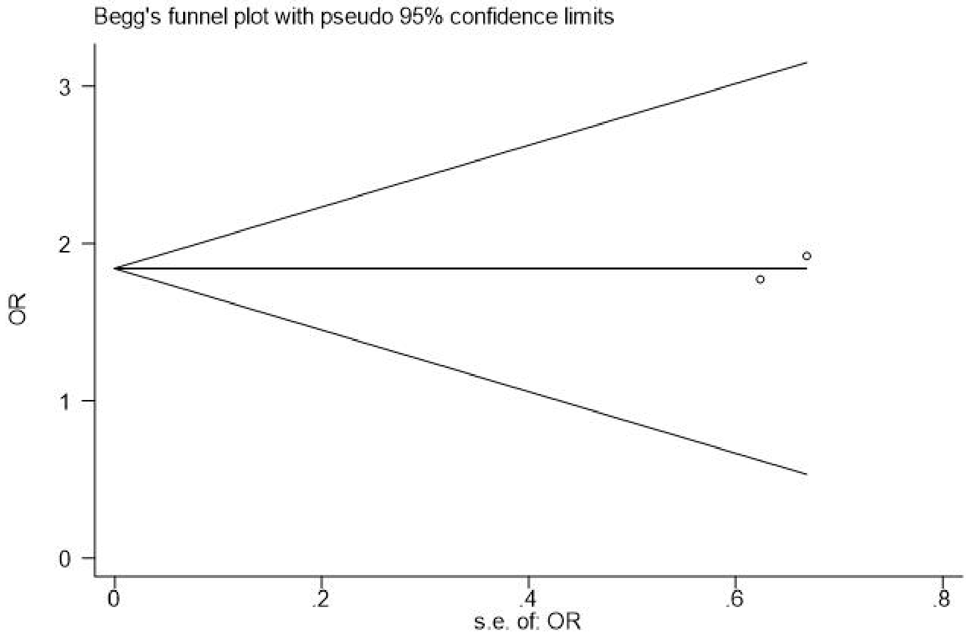


```
Begg's Test

  adj. Kendall's Score (P-Q) =        1
          Std. Dev. of Score =     1.00
           Number of Studies =        2
                          z  =     1.00
                    Pr > |z| =    0.317
                          z  =     0.00 (continuity corrected)
                    Pr > |z| =    1.000 (continuity corrected)
```

Figure 4

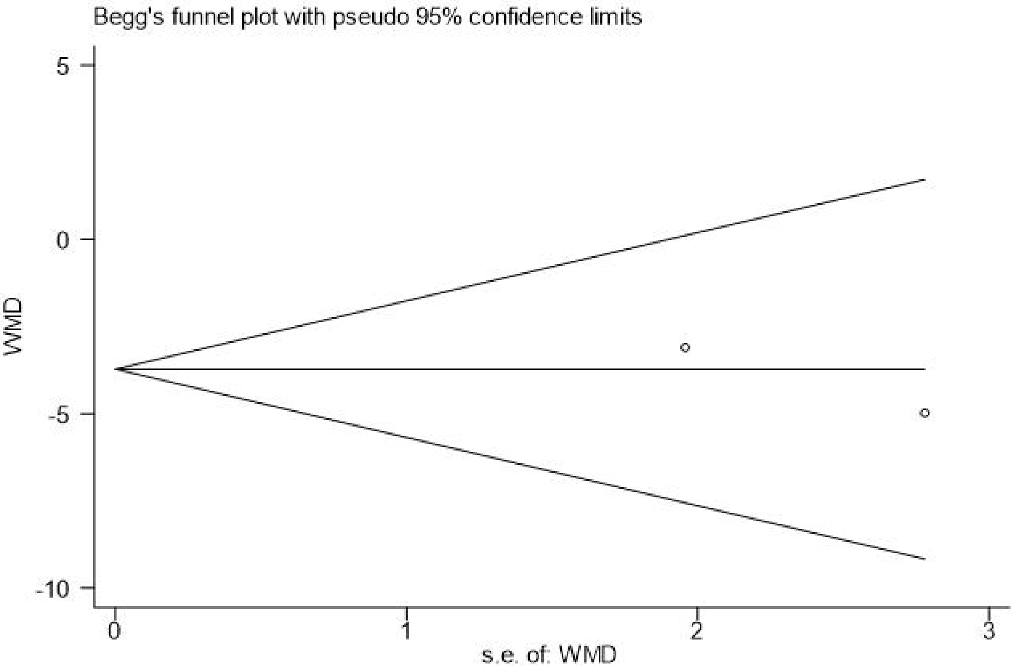


```
Begg's Test

  adj. Kendall's Score (P-Q) =        -1
          Std. Dev. of Score =      1.00
           Number of Studies =         2
                          z  =     -1.00
                  Pr > |z| =     0.317
                          z  =      0.00 (continuity corrected)
                  Pr > |z| =     1.000 (continuity corrected)
```

Figure 5

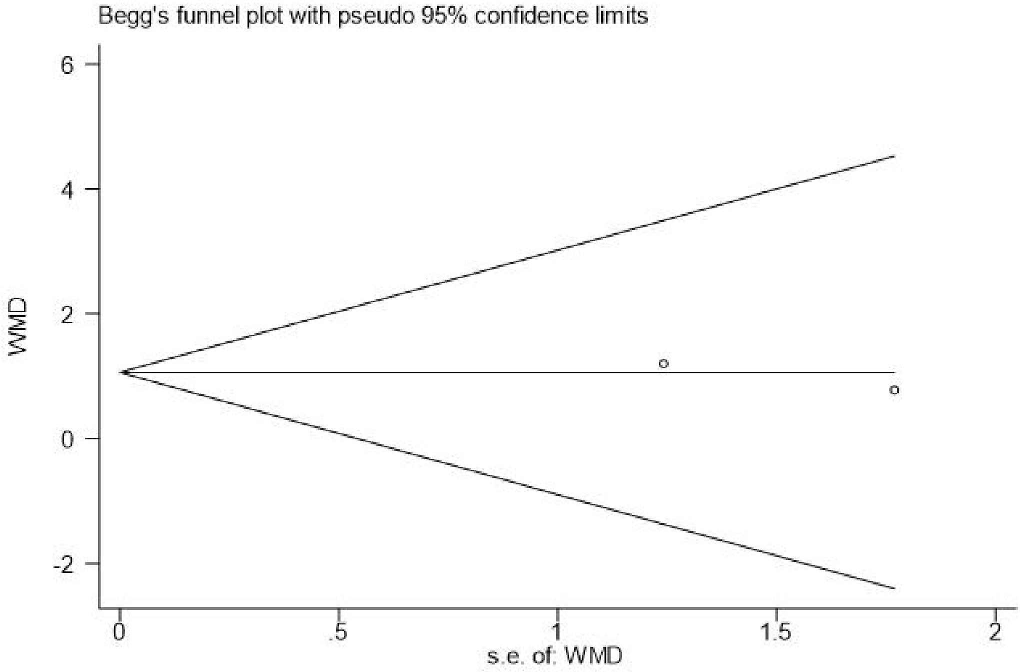


```
Begg's Test

  adj. Kendall's Score (P-Q) =      -1
          Std. Dev. of Score =    1.00
           Number of Studies =       2
                          z  =   -1.00
                    Pr > |z| =   0.317
                          z  =    0.00 (continuity corrected)
                    Pr > |z| =   1.000 (continuity corrected)
```

Figure 6

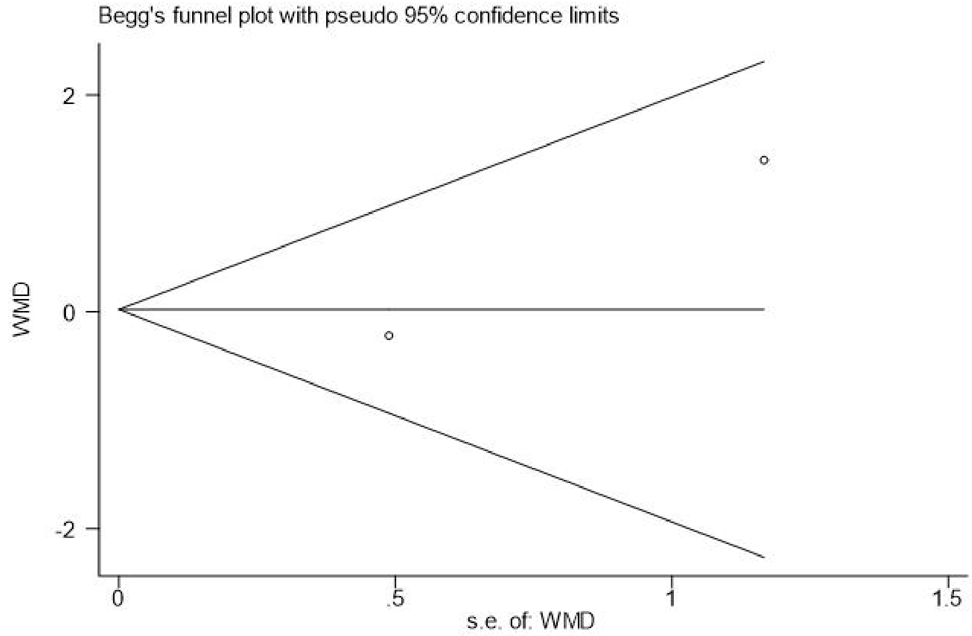


```
Begg's Test

  adj. Kendall's Score (P-Q) =        1
          Std. Dev. of Score =     1.00
           Number of Studies =        2
                         z  =     1.00
                  Pr > |z| =    0.317
                         z  =     0.00 (continuity corrected)
                  Pr > |z| =    1.000 (continuity corrected)
```

Figure 7

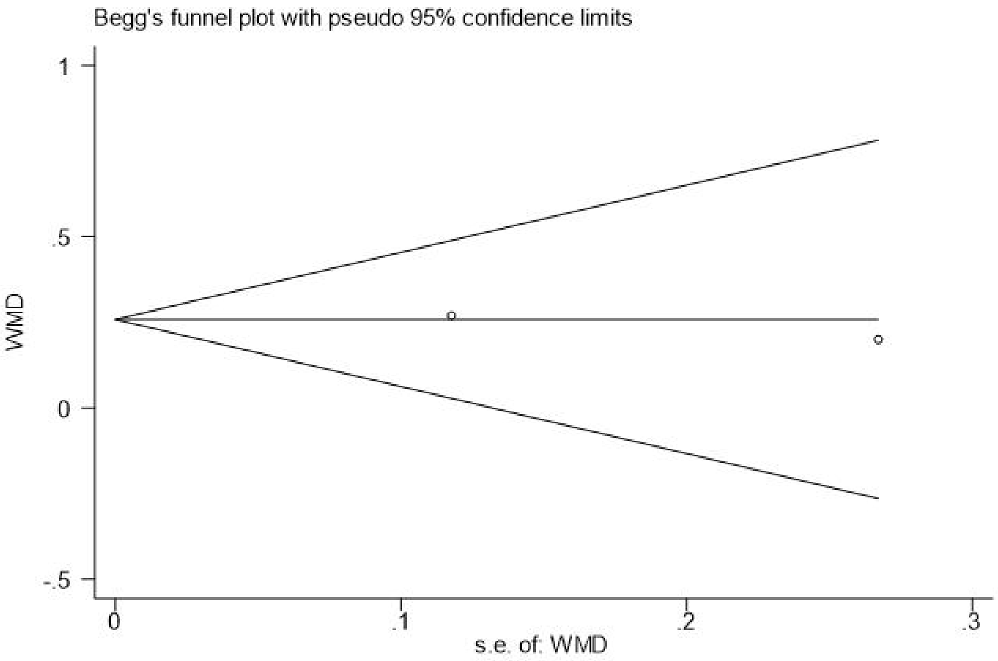


```
Begg's Test

  adj. Kendall's Score (P-Q) =        -1
          Std. Dev. of Score =      1.00
           Number of Studies =         2
                          z  =     -1.00
                  Pr > |z|  =     0.317
                          z  =      0.00 (continuity corrected)
                  Pr > |z|  =     1.000 (continuity corrected)
```
